# *Legionella pneumophila* Seropositivity-Associated Factors in Latvian Blood Donors

**DOI:** 10.3390/ijerph13010058

**Published:** 2015-12-22

**Authors:** Olga Valciņa, Daina Pūle, Irina Lucenko, Dita Krastiņa, Žanete Šteingolde, Angelika Krūmiņa, Aivars Bērziņš

**Affiliations:** 1 Institute of Food Safety, Animal Health and Environment “BIOR”, Riga LV-1076, Latvia; daina.pule@bior.lv (D.P.); dita.krastina@bior.lv (D.K.); zanete.steingolde@bior.lv (Ž.Š.); aivars.berzins@bior.lv (A.B.); 2 Centre for Disease Prevention and Control, Riga LV-1005, Latvia; irina.lucenko@spkc.gov.lv; 3 Department of Infectology and Dermatology, Riga Stradiņš University, Riga LV-1007, Latvia; angelika.krumina@rsu.lv

**Keywords:** *Legionella pneumophila*, seroprevalence, blood donors

## Abstract

Continuous environmental exposure of humans to *Legionella* may induce immune responses and generation of antibodies. The aim of this study was to investigate the seroprevalence of *Legionella pneumophila* serogroups (SG) 1–6 in the general healthy population and identify the associated host-related and environmental risk factors. *L. pneumophila* SG 1–6 seroprevalence among a total of 2007 blood samples collected from healthy donors was 4.8%. Seroprevalence was higher in women (5.9%) than men (3.3%) and in areas with a larger number of inhabitants, ranging from 3.5% in rural regions to 6.8% in the capital, Riga. Blood samples from inhabitants of apartment buildings tested positive for *L. pneumophila* in more cases (5.8%) compared to those from inhabitants of single-family homes (2.7%). Residents of buildings with a municipal hot water supply system were more likely to be seropositive for *L. pneumophila* (OR = 3.16, 95% CI 1.26–7.91). Previous episodes of fever were additionally identified as a risk factor (OR = 2.42, 95% CI 1.43–4.1). In conclusion, centralized hot water supply, female gender and previous episodes of fever were determined as the main factors associated with *L. pneumophila* seropositivity in our study population.

## 1. Introduction

Legionellosis is a form of atypical pneumonia caused by *Legionella pneumophila* and related bacteria. Clinical manifestations of the disease vary from mild influenza-like fever (Pontiac fever) to potentially lethal pneumonia (Legionnaire’s disease) [1,2]. Legionellae are ubiquitous bacteria in natural and man-made aquatic systems that can be transmitted to humans through inhalation or aspiration of contaminated water and aerosols [3]. Moreover, *Legionella* strains are able to survive in moist environments for long periods of time. Although Legionellae are widespread in man-made aquatic environments [4,5], Legionnaire’s disease is not common and mainly occurs as sporadic respiratory infections with low notification rates in the European Union, with an overall incidence of 1.1 per 100,000 inhabitants [6]. In Latvia, as of 2011, when the number of legionellosis cases increased [7], the incidence of Legionnaire’s disease has been recorded as about 1.7 cases per 100,000 inhabitants each year. Limitations in diagnostics and reporting are the main reasons underlying the lack of knowledge on the true incidence of Legionnaire’s disease and Pontiac fever [8]. Continuous environmental exposure of humans to *Legionella* triggers immune responses and the formation of antibodies, which may persist at measurable levels for several months and even years without causing any symptoms [9]. Antibodies against *Legionella* are commonly found in healthy individuals and may vary from less than 1% [10] up to 45.1% [11]. Host-related risk factors associated with Legionnaire’s disease include smoking, male sex, older age, diabetes mellitus and other underlying diseases, and the current use of medication [12,13]. Complex water distribution systems, travelling and using showers outside the home are considered environmental risk factors for legionellosis [14].

In the current study, we aimed to investigate the seroprevalence of *L. pneumophila* serogroups (SG) 1–6 in the general healthy population in Latvia and identify the associated host-related and environmental risk factors.

## 2. Experimental Section 

### 2.1. Study Population and Sampling

Overall, 2007 blood samples were collected from healthy blood donors throughout Latvia from February 2014 to October 2014 in collaboration with the Latvian State Blood Centre. Blood donors were selected based on quotes in each district based on sex and age, according to data from the 2011 Population Census [15]. Donors were unpaid healthy volunteers. Each donor was assessed by a physician, prior to blood donation, and consent to participate in the study was obtained. One out of 2008 donors refused to participate in the study. Ethical clearance and informed consent were obtained from all participants.

Participants were interviewed and asked to complete a questionnaire on residential status, self-reported health and relevant exposures over the past year. Questions regarding socio-demographic characteristics and potential exposure, including age, sex, personal habits (smoking), place, type and age of residence, type of hot water supply system and water heater, exposure to water aerosols at work such as car washes, spas, dental clinics and processing plants, showering outside the home, history of influenza-like illness, pneumonia and respiratory tract illnesses during the previous year were included.

### 2.2. Serological Methods

All 2007 samples were tested for both IgG and IgM antibodies against *Legionella pneumophila* SG 1–6 with the indirect immunoenzyme assay (Vircell, Spain). Samples with equivocal results were re-tested. According to the manufacturer, samples with indexes below nine were considered as not having antibodies against *L. pneumophila*. Samples with indexes above 11 were considered as having antibodies against *L. pneumophila.* In addition, all positive samples were specifically tested for *L. pneumophila* SG 1 using the indirect immunoenzyme assay (*Legionella pneumophila* serogroup 1 ELISA IgG, Vircell, Spain).

### 2.3. Statistical Analysis

Logistic regression analysis was performed to determine the risk factors for *Legionella* seropositivity in the blood donors. Data were stratified by the place of residence of the donors. Variables included age, sex, hot water supply system, and previous health episodes. Univariate analysis was performed for all variables in order to identify potential risk factors, which were included in the multivariate logistic regression model. Statistical analyses were performed using SPSS v.22.0 (SPSS Inc., Chicago, IL, USA).

### 2.4. Ethical Statements

All procedures performed in this work comply with the ethical standards of the relevant national and institutional committees on human experimentation and the Helsinki Declaration of 1975, as revised in 2008. All subjects gave their informed consent for inclusion before they participated in the study and the protocol was approved by the Rīga Stradiņš University Ethics Committee (Approval number: 26-4/10.35). 

## 3. Results 

Overall, *L. pneumophila* SG 1–6 seroprevalence among blood donors was 4.8% (Table 1), with 0.2% of donors being seropositive for *L. pneumophila* SG 1. Seroprevalence was higher in women (5.9%) than men (3.3%). Similar seroprevalence was observed between the 18–35 and 36–50 donor age groups (*p* > 0.05), with one exception in a group of males aged 36–50 years, but increased for the age group of 51–65 years. 

**Table 1:** Seroprevalence of *Legionella pneumophila* SG 1–6 in blood donors.

All Samples	No. Samples/Positive Samples (%)		
	Total	Females	Males
	2007/96 (4.8)	1121/67 (5.9)	886/29 (3.3)
Host-related factors			
Age group			
18–35 years	1109/51 (4.6)	584/33 (5.7)	525/18 (3.4)
36–50 years	581/27 (4.6)	354/21 (5.9)	227/6 (2.6)
51–65 years	317/18 (5.7)	183/13 (7.1)	134/5 (3.7)
Smoking			
Yes	576/18 (3.1)	219/7 (3.2)	357/11 (3.1)
No	1419/78 (5.5)	896/60 (6.7)	523/18 (3.4)
Previous health episodes			
Pneumonia			
Yes	9/1 (11.1)	3/1 (33.3)	6/0 (0.0)
No	1954/94 (4.8)	1094/65 (6.1)	860/29 (3.4)
Bronchitis			
Yes	52/3 (5.8)	38/2 (5.3)	14/1 (7.1)
No	1927/93 (4.8)	1068/65 (6.1)	859/28 (3.3)
Fever			
Yes	195/19 (9.7)	136/12 (8.8)	59/7(11.9)
No	1781/76 (4.3)	970/54 (5.6)	811/22 (2.7)
Environmental factors			
Urbanization			
Riga	615/42 (6.8)	358/34 (9.5)	257/8 (3.1)
Cities, small towns	611/27 (4.4)	342/15 (4.4)	269/12 (3.3)
Countryside	777/27 (3.5)	419/18 (4.3)	358/9 (2.5)
Type of building			
Single-family home	666/18 (2.7)	345/10 (2.9)	321/8 (2.5)
Apartment building	1320/77 (5.8)	766/56 (7.3)	554/21 (3.8)
Building age group			
built before 1950	359/8 (2.2)	196/4 (2.0)	163/4 (2.5)
built in 1951–1970	494/30 (6.1)	286/23 (8.0)	208/7 (3.4)
built 1971–1990	625/34 (5.4)	340/21 (6.2)	285/13 (4.6)
built after 1991	187/12 (6.4)	103/9 (8.7)	84/3 (3.6)
Renovation of water supply system			
Yes	752/31 (4.1)	390/19 (4.9)	362/12 (3.3)
No	936/56 (6.0)	570/42 (7.4)	366/14 (3.8)
Type of hot water preparation			
Municipal	1027/69 (6.7)	597/51 (8.5)	430/18 (4.2)
Electric heater	623/16 (2.6)	338/11 (3.3)	285/5 (1.8)
Gas heater	107/5 (4.7)	66/3 (4.5)	41/2 (4.9)
Wood-fired heating	224/5 (2.2)	112/1 (0.9)	112/4 (3.6)
Exposure at work			
Yes	92/2 (2.2)	41/0 (0.0)	51/2 (3.9)
No	1898/93 (4.9)	1073/66 (6.2)	825/27 (3.3)
Showering other than at home			
Yes	1201/65 (5.4)	654/44 (6.7)	547/21 (3.8)
No	730/26 (3.6)	424/19 (4.5)	306/7 (2.3)

Seroprevalence was higher in areas with a larger number of inhabitants, ranging from 3.5% in rural areas to 6.8% in the capital, Riga. Blood samples from inhabitants of apartment buildings were positive for *L. pneumophila* more often (5.8%) than those from inhabitants of single-family homes (2.7%). In addition, the number of positive cases varied between buildings with municipal hot water supply systems (6.7%), electric water heaters (2.6%), gas water heaters (4.7%) and wood-fired water heating (2.2%), and between those with renovated (4.1%) and non-renovated water supply systems (6.0%).

A large proportion (>60%) of the survey participants confirmed that they had taken a shower at non-residential locations outside their home (for instance gyms, hotels and hospitals) over the last year. Overall, no significant differences were evident between donors who used showers within and outside the home (*p* > 0.05). 

Only a small number of participants (4.6%) were continuously exposed to water aerosols at work, such as car washes, spas, dental clinics and processing plants. Analysis of data from the questionnaires revealed no significant differences between groups of exposed and non-exposed participants or on the influence of wearing a protective mask.

Potential risk factors for *L. pneumophila* seroprevalence were evaluated via logistic regression. Univariate analysis identified gender as a risk factor, with females being more likely to be seropositive than males (OR = 1.88, 95% CI 1.20–2.93) (Table 2). The type of residence was another risk factor, with OR = 2.23 and 95% CI 1.32–3.76 for inhabitants of apartment buildings *versus* those living in single-family homes. Donors from cities or small towns and the capital, Riga, were more likely to be seropositive than residents of rural areas (OR = 1.28, 95% CI 0.75–2.21 and OR = 2.04, 95% CI 1.24–3.34, respectively). Residents of buildings with a municipal hot water supply system were more likely to be seropositive for *L. pneumophila* (OR = 3.16, 95% CI 1.26–7.91) than residents of buildings with electric (OR = 1.16, 95% CI 0.42–3.19), gas (OR = 2.15, 95% CI 0.61–7.58) or wood-fired water heating systems. Previous fever episodes were identified as a risk factor (OR = 2.42, 95% CI 1.43–4.1), while other medical episodes, including pneumonia and bronchitis, were not associated with *L. pneumophila* seropositivity. Other potential risk factors not associated with seropositivity included the age group of donors, age of residential buildings, renovation status of the water supply systems in residential buildings, showering outside homes and aerosol exposure at work.

The risk factors identified in univariate analysis were included in the multivariate logistic regression model. The main risk factors for *L. pneumophila* seropositivity were identified as the type of hot water supply system in residential buildings, gender and previous fever episodes.

**Table 2:** Odds ratios and 95% CI for association between *Legionella pneumophila* SG 1–6 seropositivity and relevant factors.

Factor	OR	95% CI
Gender (*p* = 0.005)		
Female *vs.* Male	1.88	1.20–2.93
Residence type (*p* = 0.011)		
Apartment building *vs.* Single family home	2.23	1.32–3.76
Urbanization (*p* = 0.037)		
Riga *vs.* Countryside	2.04	1.24–3.34
Cities, small towns *vs.* Countryside	1.28	0.75–2.21
Type of hot water preparation (*p* = 0.001)		
Municipal *vs.* Wood-fired heating	3.16	1.26–7.91
Electric heater *vs.* Wood-fired heating	1.16	0.42–3.19
Gas heater *vs.* Wood-fired heating	2.15	0.61–7.58
Smoking (*p* = 0.027)		
Yes *vs.* No	0.56	0.33–0.94
Previous episodes of fever (*p* = 0.001)		
Yes *vs.* No	2.42	1.43–4.10

## 4. Discussion

Our study showed that *L. pneumophila* SG 1–6 seroprevalence depends on the degree of urbanization, ranging from an average of 3.5% in rural areas to 6.8% in Riga, the largest city in Latvia. Low seroprevalence (0.2%) of *L. pneumophila* SG 1 is consistent with our previous finding that only 19% of all environmental isolates are *L. pneumophila* SG 1, while other environmental samples are contaminated with SG 2–15 [16]. Several studies over the years have reported *Legionella* contamination in the domestic water supply system as a considerable problem [17,18,19]. Our group recently showed that 53% of apartment buildings in Riga are contaminated with *L. pneumophila* [16]. 

Limited reports on the seroprevalence of *Legionella* are documented, and the available data vary considerably. Even among countries with similar climates, geography and socio-economic factors, results of seroprevalence studies are significantly different. Earlier studies in Denmark disclosed a seroprevalence level of 22.9% in blood donors [20], while in Sweden seroprevalence was around 1.0% in healthy people [9]. In southern Europe, in Italy, seroprevalence against *L. pneumophila* SG 1–6 was 3.4% and 16.4% against *L. pneumophila* SG 7–14 [21], while in a study based in France, which included exposed and non-exposed personnel at industrial sites, the low prevalence of 2.8% was observed [22]. The main factors underlying the differences in observed *L. pneumophila* seroprevalence levels are distinct testing methods and differences in study design.

Significant differences (*p* = 0.005) in *L. pneumophila* SG 1 seroprevalence between women (5.9%) and men (3.3%) were observed, in contrast to previously defined Legionnaire’s disease risk factors [12]. Incidence data also showed the predominance of male cases [6]. The higher seroprevalence rate for Latvian women may possibly be explained due to greater exposure to water spray during household works. Women may be continuously exposed to low doses of *Legionella*, which do not cause disease but only induce immune response [7]. Moreover, women could be more resistant to Legionnaire’s disease due to the role of Toll-like receptor, which has demonstrated protective association with resistance to *Legionella* and other immunogenetic factors [23]. A significantly higher male-to-female ratio of patients with *L. pneumophila* SG 1 was reported compared to those with other *L. pneumophila* serogroups in Japan [24].

An interesting association was observed between smoking and seropositivity. According to our results, seroprevalence among smoking women was lower than that among non-smokers. Only 3.2% of smoking female donors were *L. pneumophila* possibly seropositive, while among non-smoking females, seropositivity reached 6.7% (OR = 0.46). It could be associated with the suppressive effect of smoking on the protective functions of humoral immunity. As reported in previous studies, tobacco smoking was associated with lower levels of IgG [25]. This finding can be explained by the limitations of our study population, since the majority of donors represented healthier individuals and this study did not specifically focus on the distribution of smoker groups.

Data from previous health episodes did not indicate blood donors with a history of Legionnaire’s disease. Similarly, no significant differences were observed for donors with and without indications of pneumonia or bronchitis episodes in the previous year. However, we observed significant differences (OR = 2.42, *p* = 0.001) for donors of both sexes and all age groups suffering from a flu-like disease over the last year, which could indicate underdiagnosed Pontiac fever cases due to limitations in diagnostics [26].

Type of residence, degree of urbanization and type of hot water preparation system were the most significant environmental factors identified in our study. Residents of apartment-style houses were at greater risk than those living in single-family homes (OR = 2.23, *p* = 0.011). Moreover, areas with higher population density were associated with higher odds of seropositivity (OR = 1.89, *p* = 0.046). Similarly, residents of buildings that received municipal hot water and were unable to influence either hot water temperature or overall condition of the water supply system were at the greatest risk of seropositivity (OR = 3.16, *p* = 0.001). Notably, the type of hot water preparation had the most significant effect in multivariate logistic regression analysis. These findings are consistent with results of previous environmental studies, where *Legionella* prevalence was higher in buildings with centralized hot water supply systems [27].

We obtained no evidence on whether the age of the building affects seroprevalence results. More than 67% of the participants lived in buildings constructed between the 1950s and 1990s, and renovation of the water supply systems was not carried out in most cases. However, previous studies have reported an association between the number of different predominant *Legionella* genotypes and the age of the building of residence [28].

## 5. Conclusions

Municipal hot water supply system, female gender and previous episodes of fever were determined as the main factors associated with *L. pneumophila* seropositivity. Building management plays a crucial role in facilitating preventive actions against contamination in apartments. Building managers ensure the disinfection of the water system in buildings and the maintenance of the appropriate circulation temperature. However, the low economic status in some countries necessitates situations whereby the water temperature is voluntarily reduced due to the decision of the residents to save hot water supply costs. Effective strategies for preventing legionellosis need to involve education of both the community and building managers, establishment of risk-based reference values for *Legionella* in water, and careful choice of clinical diagnostic methods related to environmental and clinical findings. 
